# Analysis of Reasons for Permanent Teeth Extraction in Dental College Teaching Hospital of Mustansiriyah University, Baghdad/Iraq: A Prospective Cross‐Sectional Observational Study

**DOI:** 10.1155/ijod/2713944

**Published:** 2026-05-26

**Authors:** Falah Hasan Jumaah

**Affiliations:** ^1^ Department of Oral Surgery and Periodontology, College of Dentistry, Mustansiriyah University, Baghdad, Iraq, uomustansiriyah.edu.iq

**Keywords:** dental caries, periodontal disease, tooth extraction

## Abstract

**Introduction:**

Tooth extraction represents a global concern, as it directly affects the quality of life. Despite of advancements in modern dentistry, extraction remains the only available option in not a few cases. The most common causes of tooth loss are caries and periodontal diseases, with other less common causes like trauma, orthodontic treatment, and patient preference in low socioeconomic areas. This study aims to ascertain the frequency, causes, and patterns of adult tooth extractions among Iraqi patients.

**Materials and Methods:**

Patients attending the clinics of oral surgery in the dental teaching hospital of Mustansiriyah University for the period of October 2024 to May 2025 were examined to be enrolled in this study with their information recorded and analyzed.

**Results:**

A total of 1096 patients participated in this study with 1408 extracted teeth. Almost 738 were males, and 358 were females with an age range of (18–83) years with a mean ± Sd of (40.3 ± 14.34) years. Dental caries was found to be the most common cause of tooth extraction (94.5%), and periodontal disease accounted for (3.8%) of extractions; other causes represented only (1.6%). No statistically significant association was observed between age group and extraction due to caries (*χ* ^2^ = 3.35, *p* = 0.067; OR = 0.62, 95% CI: 0.36–1.06). In contrast, a significant association was found between age group and extraction due to periodontal disease (*χ* ^2^ = 10.53, *p* = 0.001 OR = 3.00, 95% CI: 1.47–6.13). The association between age, gender, and systemic disease with tooth extraction due to caries was tested by binary logistic regression analysis and showed that systemic disease was significantly associated with the outcome. Patients with systemic disease had lower odds of tooth extraction due to caries compared with those without systemic disease (OR = 0.32, 95% CI: 0.17–0.60, *p*  < 0.001). Age (OR = 1.00, 95% CI: 0.98–1.02, *p* = 0.969) and gender (OR = 0.75, 95% CI: 0.42–1.34, *p* = 0.332) were not significantly associated with caries‐related tooth extraction.

**Conclusion and Suggestions:**

Dental caries was the predominant cause of permanent tooth extraction among patients attending the Dental College Teaching Hospital of Mustansiriyah University, accounting for the majority of cases, while periodontal disease represented a considerably smaller proportion. No statistically significant association was observed between age group and extraction due to caries. In contrast, a significant association was found between age group and extraction due to periodontal disease. These findings highlight the need for effective preventive strategies and patient education programs aimed at improving oral hygiene practices and early management of dental caries to reduce the rate of tooth loss.

## 1. Introduction

Tooth loss is one of the most complicated dental concerns all over the world [1]. Status of oral health and life quality are directly affected by permanent teeth loss. Partial or complete edentulism is directly linked to a multitude of metabolic and dental diseases; tooth loss is considered as the primary indicator of utilization or access to dental care [2]. Historically, dental caries and periodontal disease have been identified as primary contributors to tooth loss globally [3]. Indeed, untreated dental caries can lead to progressive health risks, including pain, infection, and eventual tooth loss, thereby burdening individuals and healthcare systems [4]. Similarly, periodontal disease, a pervasive inflammatory condition affecting the supporting structures of the teeth, remains a major etiology for tooth extraction, often leading to tooth mobility and eventual exfoliation if left untreated [5]. Despite advances in conservative dental practices, which prioritize tooth retention, extraction frequently remains the sole viable treatment option in cases where the progression and severity of these oral diseases are advanced [6]. Beyond these well‐established factors, the global burden of oral diseases, encompassing dental caries, periodontal disease, and other conditions, highlights the persistent challenges in maintaining optimal oral health worldwide [7]. Consequently, a detailed examination of the multifactorial etiologies driving tooth extraction is crucial for developing effective preventive strategies and improving clinical outcomes.

In the literature, the main reason for tooth extraction varied among different studies, with dental caries being the most frequent and periodontal diseases as the second most frequent; other reasons mentioned in the literature were trauma, orthodontic, and periapical diseases.

Despite the significant public health implications of tooth loss, there is a scarcity of recent data describing the patterns and causes of tooth extraction in Iraq. Therefore, this study aimed to analyze the reasons for permanent tooth extraction among patients attending the Dental College Teaching Hospital of Mustansiriyah University in Baghdad, Iraq, and to assess the association between tooth extraction and selected demographic and clinical variables, including age, gender, and systemic diseases. It was hypothesized that dental caries would represent the leading cause of tooth extraction and that demographic and clinical factors such as age, gender, and systemic diseases would be significantly associated with tooth loss.

Understanding the primary causes of tooth loss in this population may help inform preventive strategies, improve oral health awareness, and support the development of targeted dental health program and to provide information and instruction to various Iraqi societies, particularly the most affected segments of the community, with an emphasis on the reason for the extraction, it is necessary to identify which age group, gender, and tooth position have the highest extraction percentage.

## 2. Materials and Methods

### 2.1. Study Design and Location

This *prospective cross-sectional observational study* is conducted in the clinics of the Department of Oral Surgery and Periodontology/the Teaching hospital of the Dental College of Mustansiriyah University, for duration of 8 months (from October 2024 to May 2025).

This study was reported in accordance with the STROBE guidelines [8], and the completed checklist is available in the Supporting Information.

Potential sources of bias, including selection bias related to the teaching hospital setting and refusal bias, were considered during study design and interpretation.

### 2.2. Ethical Approval

This study was conducted in accordance with the ethical principles of the Declaration of Helsinki and approved by the Research Ethics Committee of Mustansiriyah University, College of Dentistry (Study N MUOSU‐202123).

### 2.3. Population and Sample

Patients attending the department of Oral Surgery and Periodontology clinics in the teaching hospital of the dental college in the time period from October 2024 to May 2025 participated in this study with the inclusion criteria of:

• Age of 18 years or older.
• Patients needing extraction of 1 or more teeth.
• Patients agreeing to participating in this study (signing an informed consent).
• The present study primarily included permanent teeth for the assessment of extraction patterns and underlying causes. However, cases of retained deciduous teeth were also identified within the study population, particularly in older individuals exhibiting prolonged retention. Such teeth were not excluded; instead, they were recorded and analyzed where clinically relevant. The inclusion of retained deciduous teeth was based on their presence at the time of examination and their indication for extraction. Clear differentiation between permanent and retained deciduous teeth was maintained during data collection to ensure accuracy and consistency in the analysis.

Exclusion criteria were:

• Any patient who didn’t approve to participate in this study.
• Third molar teeth extraction was excluded. (Third molars were excluded from the analysis because they are frequently removed for prophylactic, orthodontic, or impaction‐related reasons and may not represent tooth loss due to pathological dental conditions.)
• Records with incomplete information regarding the variables of interest were excluded from the analysis. Only cases with complete data were included in the final dataset used for statistical evaluation.

The sample size consisted of all patients who underwent permanent tooth extraction at the Dental College Teaching Hospital during the study period and met the study inclusion criteria. As this was an observational study based on consecutive cases, a formal sample size or power calculation was not performed.

Prior to data collection, the examiners were trained and calibrated to ensure consistency and reliability in the clinical assessment and recording of tooth extraction data.

Each patient was examined clinically to determine if extraction is the last line for treatment, and if the tooth is restorable, the patient was referred to conservative department clinics. To confirm the existence of periapical, periodontal, or general bony abnormalities, some patients required additional investigation, such as periapical or panoramic radiography. Clinical findings from the examination and investigation, as well as the patient’s medical and dental history, were documented in the case sheet. Every extraction was carried out under lidocaine local anesthetic 2% (1:100,000).

Extractions were classified according to their primary cause. Caries‐related extraction was defined as extraction due to extensive carious destruction of the tooth that rendered it nonrestorable or associated with pulpal or periapical pathology. Periodontal‐related extraction was defined as extraction resulting from advanced periodontal disease characterized by severe attachment loss, marked tooth mobility, or significant alveolar bone loss leading to a poor prognosis for the tooth.

### 2.4. Variables

A number of variables were investigated, including age, gender, systemic disease status, the reason for tooth extraction, tooth position, and number of teeth needing extraction.

### 2.5. Data Analysis

The collected data were analyzed using GraphPad Prism (Version 10) Windows software (GraphPad software, CA, USA). The descriptive statistics include percentages, or mean ± standard deviation. The statistical analysis in this study was conducted using a confidence level of 95%, with a corresponding significance level (*α*) set at 0.05. The included variables were analyzed using Shapiro–Wilk test of normality, *χ* ^2^ test were used to assess the association between different variables. Binary logistic regression was used to adjust for potential confounders, including age, gender, and systemic disease.

This study was reported in accordance with the ethicBE (Strengthening the Reporting of Observational Studies in Epidemiology) guidelines.

## 3. Results

A total of 1643 patients were received in oral surgery clinics for the time period mentioned earlier. A total of 324 of them refused to participate in the study, and 223 patients were not indicated for extraction and referred to other clinics. So, a total of 1096 patients participated in this study with 1264 extracted teeth.

Of the 1096 patients, 738 were males, and 358 were females (see Table 1), with a male to female ratio of 369: 179 (there are about 2.06 males for every 1 female). The age range of the patients was (18−83) years with a mean ± Sd of (40.3 ± 14.34) years. According to age, the patients were subdivided into two groups (≥40 and <40 years) based on previous literature and clinical relevance (see Table 1):

**Table 1:** Demographic and clinical characteristics of the study population and distribution of tooth extractions.

Gender		Total
Male	738 (67.3%)	1096
Female	358 (32.6%)	
Age		
≥40 years	576 (52.5%)	1096
<40 years	520 (47.5%)	
Extracted teeth		
Single tooth extraction	906 (64.3%)	1096
Multiple teeth extraction	190 (35.6%)	
Multiple teeth extraction distribution		
Two teeth extracted	126 (66.3%)	190
Three teeth extracted	38 (20%)	
Four teeth extracted	10 (5.2%)	
More than four teeth extracted	16 (8.4%)	
Systemic disease status		
Patients with no systemic disease	928 (84.6%)	1096
Patients with systemic disease	168 (15.3%)	
Systemic disease distribution		
Hypertension	54 (32.14%)	168
Diabetes mellitus	40 (23.8%)	
Hypertension + diabetes	8 (4.76%)	
Hypertension + other diseases	36 (21.42%)	
Mucocutaneous	4 (2.38%)	
Stroke	4 (2.38%)	
GIT diseases	8 (4.76%)	
Thyroid disease	14 (8.33%)	
Reason for extraction		
Dental caries	1036 (94.5%)	1096
Periodontal disease	42 (3.8%)	
Others	18 (1.6%)	
Dental caries subgroups		
Painful extracted because nonrestorable	758 (73.1%)	1036
Painless extracted for prosthodontic treatment	278 (26.8%)	
Other reasons for extraction	—	—
Trauma	6 (33.3%)	18
Malposed	2 (11.1%)	
Orthodontic	10 (55.5%)	
Extraction frequency by tooth group		
Molar	540	1264
Premolar	496	
Anterior	228	

*Note:* Description as frequency and percentages.

• ≥40 years: presented as 576 (52.5%) of the sample.
• <40 years: presented as 520 (47.4%) of the sample.

A total of 906 of the patients had single tooth extraction (64.3%), while 190 patients had multiple extractions (two or more teeth extracted) and represented 35.6% of the sample (Table 1). The mean number of extractions per patient was 1.28.

Of the 1096 patients, 928 (84.6%) were healthy with no systemic diseases, and 168 (15.3%) had one or more systemic diseases, as shown in Table 1.

Analysis of the reason for tooth extraction revealed that dental caries is the most common cause of tooth extraction among Iraqi patients attending the teaching hospital of Mustansiriyah University, and periodontal disease represented the second common cause for extraction; other causes represented only 1.6% as shown in Table 1.

Further subgroup analysis was performed to evaluate the distribution of caries‐related extractions according to demographic variables. The analysis showed that there was no statistically significant association between age group and extraction due to caries (*χ* ^2^ = 3.35, *p* = 0.067; OR = 0.62, 95% CI: 0.36–1.06). Similarly, the distribution of caries‐related extractions across gender (*χ* ^2^ = 0.20, *p* = 0.65. OR = 0.88; 95% CI: 0.50–1.55), and systemic disease status (*χ* ^2^ = 16.15, *p* < 0.001. OR = 3.0; 95% CI: 1.73–5.21) did not show statistically significant differences (Tables 2 and 3).

**Table 2:** Subgroup analysis of dental caries‐related tooth extractions according to gender and systemic disease status.

Association of gender group with extraction due to caries					
Group	Extraction due to caries		Not due to caries	Total	*χ* ^2^ test
Males	696		42	738	*χ* ^2^ = 0.20
Females	340		18	358	*p* = 0.65 (N.S)
Total	1036		60	1096	OR = 0.88; 95% CI: 0.50–1.55

**Association of patient’s systemic disease status with extraction due to caries**					

Patients without systemic diseases	888		40	928	*χ* ^2^ = 16.15
Patients with systemic diseases	148		20	168	*p* = 0.001 ^∗^
Total	1036		60	1096	OR = 3.0; 95% CI: 1.73–5.21

^∗^Significant.

**Table 3:** Association of age group with reason of extraction (caries and periodontal disease).

Association of age group with extraction due to caries				
Group	Extraction due to caries	Not due to caries	Total	*χ* ^2^ test
≥40	539	37	576	*χ* ^2^ = 3.35
<40	500	20	520	*p* = 0.067
Total	1036	60	1096	OR = 0.62, 95% CI: 0.36–1.06

Association of age with periodontal disease				
Group	Extraction due to periodontal disease	Not due to periodontal disease	Total	*χ* ^2^ test
≥40	32	544	576	*χ* ^2^ = 10.53
<40	10	510	520	*p* = 0.001 ^∗^
Total	42	1050	1096	OR = 3.00, 95% CI: 1.47–6.13

^∗^Significant.

The association between age group and reasons for tooth extraction was assessed using the *χ* ^2^ test and odds ratio analysis. No statistically significant association was observed between age group and extraction due to caries (*χ* ^2^ = 3.35, *p* = 0.067; OR = 0.62, 95% CI: 0.36–1.06). In contrast, a significant association was found between age group and extraction due to periodontal disease (*χ* ^2^ = 10.53, *p* = 0.001). Patients aged ≥40 years had three times higher odds of periodontal‐related tooth extraction compared with those aged <40 years (OR = 3.00, 95% CI: 1.47–6.13) (Table 3).

No statistically significant difference was observed between upper and lower teeth extraction frequencies (*χ* ^2^ = 2.9, df = 1, *p* = 0.088), despite a slightly higher number of extractions occurring in the upper teeth. The distribution of extracted teeth was divided into three groups: molars, premolars, and anterior (Table 1). A *χ* ^2^ test was performed to compare the frequency of extractions among different tooth groups (molars, premolars, and anterior). The results showed a statistically significant difference in extraction distribution between the three groups, *χ* ^2^ (2) = 135.1, *p* < 0.0001. Molars were the most frequently extracted teeth (*n* = 540), followed by premolars (*n* = 496), while anterior were extracted considerably less often (*n* = 228). This distribution indicates that molars, particularly the first molars, are disproportionately affected compared to other tooth groups.

Of the 1096 patients, 738 (67.3%) were males and 358 (32.7%) were females. A *χ* ^2^ test showed that this distribution significantly deviated from an expected equal distribution, *χ* ^2^ (1) = 131.7, *p* < 0.0001. This indicates a significantly higher proportion of male patients compared to female patients.

Binary logistic regression analysis was performed to evaluate the association between age, gender, and systemic disease with tooth extraction due to caries. The model showed that systemic disease was significantly associated with the outcome. Patients with systemic disease had lower odds of tooth extraction due to caries compared with those without systemic disease (OR = 0.32, 95% CI: 0.17–0.60, *p* < 0.001). Age (OR = 1.00, 95% CI: 0.98–1.02, *p* = 0.969) and gender (OR = 0.75, 95% CI: 0.42–1.34, *p* = 0.332) were not significantly associated with caries‐related tooth extraction (males had lower odds of extraction due to caries compared with females, but the association was not statistically significant, as shown in Table 4).

**Table 4:** Logistic regression results.

Variable	Odds ratio (OR)	95% CI	*p*‐Value
Age	1.00	0.98–1.02	0.969
Gender	0.75	0.42–1.34	0.332
Systemic disease	0.32	0.17–0.60	<0.001

## 4. Discussion

Tooth extraction is a common dental procedure performed when saving a natural tooth is no longer feasible or would threaten a patient’s oral health. Reasons for extraction range from extensive decay or infection that endangers surrounding tissues to irreversible damage from trauma, severe periodontal disease, and nonviable or impacted teeth that disrupt function or space management [9]. In addition, orthodontic crowding and third molar (wisdom tooth) removal are frequent indications when alignment or potential future complications are anticipated [10]. While contemporary dentistry emphasizes tooth preservation, extractions remain a prudent option when conservative treatments are unlikely to succeed, would fail to relieve pain, or could compromise systemic health or the longevity of adjacent teeth [11]. Understanding the indications, procedural steps, and aftercare helps patients participate in shared decision‐making with their clinicians and supports a smoother recovery. As with any surgical procedure, informed consent, assessment of infection risk, and consideration of alternative therapies are essential components of optimal care [12].

The findings of the present study provide important epidemiological data regarding the causes of tooth extraction in this population and may contribute to improving preventive dental strategies aimed at reducing tooth loss.

Research across different regions consistently highlights dental caries and periodontal disease as the primary causes for tooth extraction. In this study, caries was the primary reason for tooth extraction followed by periodontal disease. In the age group (≥40) caries was the main reason for extraction, while periodontal disease was the main reason for extraction in the age group (<40). The most prevalent tooth extracted was the first molar in both arches. This is in agreement with other research [13–15].

The findings of the present study showed that dental caries was responsible for the majority of tooth extractions (94.5%). The extremely high proportion observed in this study warrants critical interpretation within the regional context. This finding may reflect underlying socioeconomic challenges, including limited access to preventive dental care, lower oral health awareness, and financial constraints that delay treatment‐seeking behavior until extraction becomes the only viable option. Evidence suggests that dental caries disproportionately affects socially disadvantaged populations, with income, education, and occupation significantly associated with higher risk of untreated carious lesions [16]. In developing and transitional healthcare systems, inequalities in access to oral healthcare services remain a major determinant of disease burden, often resulting in delayed presentation and advanced stages of caries requiring extraction [17].

Furthermore, global data indicate that dental caries is a widespread public health problem affecting billions of individuals, with higher prevalence observed in regions with limited preventive programs and weaker healthcare infrastructure [18]. Studies from Middle Eastern and developing countries have reported persistently high caries prevalence despite availability of services, suggesting that utilization patterns, awareness, and health‐seeking behavior play a critical role [19]. Additionally, healthcare systems in many low‐ and middle‐income countries tend to emphasize curative rather than preventive care, further contributing to the progression of untreated carious lesions [20].

Furthermore, as the study was conducted in a dental teaching hospital where many patients present with advanced dental conditions, the proportion of extractions due to caries may appear higher compared with community‐based studies.

Collectively, these factors may explain the markedly high rate of caries‐related extractions observed in the present study and highlight the urgent need for strengthening preventive strategies, improving accessibility to conservative dental treatments, and implementing community‐based oral health education programs to reduce the burden of avoidable tooth loss.

A study in Japan reported that caries and its sequelae accounted for 43.3% of extractions, while periodontal disease was responsible for 41.8%. Similarly, a systematic review suggests that caries is implicated in 36.0%–55.3% of extractions and periodontitis in 24.8%–38.1% [21].

Age plays a significant role in the prevalence of these conditions as reasons for tooth loss. Younger populations tend to lose teeth primarily due to caries, while older individuals are more likely to experience tooth loss due to periodontal disease [13]. For example, in Kuwait, caries was found to be the principal cause of tooth loss in individuals aged 40 and below, whereas periodontal disease predominated in those aged 40 and above [13]. In the present study, no statistically significant association was found between age group and extraction due to caries. Similar findings were reported by Kumar et al. [22], who found no statistically significant association between age and the causes of tooth extraction, despite dental caries being the most frequent reason for extraction.

McCaul et al. [23] reported that dental caries remained the most common reason for tooth extraction across different patient groups, indicating that demographic factors such as age may not always significantly influence caries‐related extractions.

Previous studies have also shown that dental caries remains the predominant cause of tooth extraction and may occur across various age groups [24–26].

Beyond dental health issues, extractions are also carried out for a variety of other reasons, including orthodontic purposes, trauma, and patient preferences. For instance, a study in South Wales found that 5.5% of extractions were performed for orthodontic reasons [27]. Additionally, occasional extractions occur due to nondental reasons such as financial considerations or cultural beliefs, although these are not as frequently documented [21].

In Scotland, a study revealed that extraction was most commonly performed for dental caries reasons (51%), with periodontal and orthodontic being the other common causes with a percentages of 21% and 11% respectively. Other causes were failed RCT (4%), trauma, pericoronitis, and others (5.5%). The group of 40 years of age or younger was the most prevalent age group having extraction, with 54% of total extractions performed in this study, while the age group of over 60 accounted for 13.6% of all extractions [28].

A study by [29] in Saudi Arabia revealed that in all age groups, dental caries, along with its sequels, was the main reason for tooth extraction, with the highest extraction percentage in the age group of (30−39 years), and the lowest in (10−19 years) age group. Females performed more extractions than males in all of the age groups, except for the group of above 60 years. The most commonly extracted tooth was the first molar in both arches.

In Iran a study by [30] revealed that the highest age group performed extraction was the 41−60 years old (36.9%). A total of 48.7% of the patients were males, and 51.3% were females. Despite female predominance, males had more extractions than females (1470 and 1150 teeth, respectively). Caries was the main reason for tooth extraction (51%), followed by periodontal disease and impacted/supernumerary teeth (14.4% and 13.9% respectively). A significant association was computed between number of extracted teeth and characteristics of the patient (age, sex, and level of education).

Another study in Iran reported that the prevalence of tooth loss and edentulism was significantly higher among males compared with females (*p* < 0.0001). Current smoking and the presence of chronic systemic conditions, including diabetes mellitus, hypertension, and hyperlipidemia, were significantly associated with a greater number of missing teeth (*p*  < 0.0001). Moreover, individuals with lower educational attainment were more likely to exhibit higher levels of tooth loss. Statistically significant differences were also observed among tooth loss categories with respect to age and body mass index (BMI), as individuals with more extensive tooth loss tended to be older and demonstrated higher BMI values (*p* < 0.0001) [31].

A 4‐year follow‐up study in Brazil showed that over a 4‐year period, 35.7% of adults experienced tooth loss, with a mean incidence of 0.91 teeth lost per individual. Tooth loss was significantly more common in older adults, although the number of teeth lost among affected individuals was similar between younger and older adults [6].

The findings of the present study have important and specific public health implications. The overwhelmingly high proportion of extractions due to dental caries (94.5%) clearly indicates that caries remains the dominant unresolved oral health problem in this population, reflecting a substantial gap in preventive and early intervention services. This pattern is consistent with evidence showing that dental caries remains the most prevalent oral disease globally and a leading cause of tooth loss, particularly in low‐ and middle‐income countries, where preventive services are limited [32, 33]. The observed reliance on extraction as a primary treatment approach suggests that patients often seek care at advanced stages of disease, a pattern commonly associated with limited access to dental services and low oral health awareness [34].

Patients with systemic diseases had lower odds of tooth extraction due to caries compared with those without systemic diseases (OR = 0.32, 95% CI: 0.17–0.60, *p* < 0.001). Although the sample sizes differ between individuals with and without systemic diseases, the observed association may reflect underlying behavioral, medical, or care‐related factors that influence oral health outcomes. For instance, patients with systemic conditions might receive more frequent medical or dental supervision, adopt stricter oral hygiene practices, or have dietary restrictions that reduce caries risk.

A large electronic health record study in an elderly population showed that patients with systemic diseases (like diabetes, rheumatoid arthritis [RA], dementia, and hypertension) had significantly higher prevalence of dental disease and tooth loss compared with those without systemic conditions. The authors specifically noted that tooth loss was much more common in patients with diabetes and RA, suggesting that systemic disease increases risk of outcomes that often lead to extraction (e.g., caries and periodontal breakdown) [35]. In a comparative study of patients with RA versus controls, researchers found that RA patients had more teeth missing due to caries (average ~8.3 teeth) than in the control group (~5.5 teeth), although the difference did not reach conventional statistical significance (*p* ~ 0.06). This still points to a trend where a systemic inflammatory disease group exhibited more teeth lost from caries [36]. Multiple clinical studies summarized in systematic reviews show that patients with diabetes (especially poorly controlled) tend to have a higher burden of dental caries and tooth loss compared with people without diabetes. Many of these losses result from untreated caries progressing to the point of extraction in real‐world settings [37].

Consequently, there is a critical need to prioritize population‐based preventive strategies, including community‐based oral health promotion, school‐based interventions, and early detection programs targeting high‐risk individuals. Previous studies have demonstrated that preventive measures such as fluoride exposure, oral health education, and regular dental visits significantly reduce the incidence and progression of dental caries [38, 39]. Moreover, the relatively lower contribution of periodontal disease compared to caries suggests that public health efforts in this setting should be primarily focused on caries prevention and management.

The data also highlight potential inequalities in access to dental care, supporting the need for integrating oral health into primary healthcare systems to improve early diagnosis and reduce disease burden. Strengthening health systems toward preventive and minimally invasive care has been recommended as a key strategy to reduce avoidable tooth loss and improve population oral health outcomes [7, 33]. Overall, the present findings provide evidence to support targeted, data‐driven public health planning aimed at shifting from extraction‐based care toward prevention‐oriented and tooth‐preserving approaches.

The findings may be generalizable primarily to similar hospital‐based populations in Iraq and comparable healthcare settings.

### 4.1. Limitations

This study has several limitations that should be acknowledged. First, the single‐center design may limit the generalizability of the findings, as data were collected from only one dental teaching hospital. Patients attending a teaching institution may differ from the general population in terms of disease severity, access to care, and health‐seeking behavior, thereby introducing potential selection bias.

In addition, the exclusion of third molars may have influenced the reported distribution and causes of tooth extraction, as these teeth are commonly extracted for reasons that differ from other permanent teeth, such as impaction or pericoronitis.

Another important limitation is the lack of assessment of potential confounding variables, including socioeconomic status, smoking habits, and educational level. These factors are well known to influence oral health status and patterns of tooth loss, and their omission may have affected the observed associations and limited the ability to control for confounding effects.

Regarding sample size, all eligible patients who underwent permanent tooth extraction during the study period were included. As this was an observational study based on consecutive case recruitment, a formal sample size or power calculation was not performed, which may affect the statistical robustness of the findings.

Finally, the study may be subject to refusal bias, as 324 patients declined participation. Individuals who refused to participate may differ systematically from those who consented in terms of demographic or clinical characteristics, potentially affecting the representativeness of the sample and introducing bias into the results.

## 5. Conclusion

In conclusion, dental caries was the predominant reason for permanent tooth extraction in the studied population, accounting for the vast majority of cases (94.5%), while periodontal disease represented a considerably smaller proportion. Statistical analysis showed no significant association between gender and caries‐related tooth extraction, whereas a significant association was observed with systemic disease status. Additionally, no statistically significant difference was found between upper and lower tooth extraction frequencies. These findings emphasize the importance of strengthening preventive strategies, improving oral health education, and promoting early management of dental caries to reduce the burden of tooth loss.

## Author Contributions

**Falah Hasan Jumaah**: conceptualization, methodology, formal analysis, investigation, resources, data curation, writing – original draft, writing – review and editing, visualization, project administration.

## Funding

No funding was received for this research.

## Conflicts of Interest

The author declares no conflicts of interest.

## Supporting Information

Additional supporting information can be found online in the Supporting Information section.

## Supporting information


**Supporting Information** This study was designed, conducted, and reported in accordance with the Strengthening the Reporting of Observational Studies in Epidemiology (STROBE) guidelines for observational research. The STROBE checklist was used to ensure comprehensive and transparent reporting of all relevant aspects of the study design, methodology, analysis, and results. A completed STROBE checklist is provided as a supporting file (STROBE checklist.docx) to facilitate the assessment of reporting quality and adherence to recommended standards [8].

## Data Availability

The data that support the findings of this study are available from the corresponding author upon reasonable request.
