# Supplementary material for: Analysis of Reasons for Permanent Teeth Extraction in Dental College Teaching Hospital of Mustansiriyah University, Baghdad/Iraq: A Prospective Cross‐Sectional Observational Study
Source: Int J Dent. 2026 May 26;2026:2713944. doi: 10.1155/ijod/2713944 (PMC13212041; doi:10.1155/ijod/2713944)
Supplement: Supplementary file 1 — Supporting Information This study was designed, conducted, and reported in accordance with the Strengthening the Reporting of Observational Studies in Epidemiology (STROBE) guidelines for observational research. The STROBE checklist was used to ensure comprehensive and transparent reporting of all relevant aspects of the study design, methodology, analysis, and results. A completed STROBE checklist is provided as a supporting file (STROBE checklist.docx) to facilitate the assessment of reporting quality and adherence to recommended standards [8]. [file IJOD-2026-2713944-s001.docx]

**STROBE Checklist for Cross-Sectional Studies**

**Title of Study:** Analysis of Reasons for Permanent Teeth Extraction in Dental College Teaching Hospital of Mustansiriyah University, Baghdad/Iraq: A Prospective Cross- Sectional Observational Study

**Title and Abstract**

| Item No. | Recommendation | Reported on Page No. |
| --- | --- | --- |
| 1a | Indicate the study design with a commonly used term in the title or abstract | Title / Abstract |
| 1b | Provide an informative and balanced summary of what was done and what was found | Abstract |

**Introduction**

| Item No. | Recommendation | Reported on Page No. |
| --- | --- | --- |
| 2 | Explain the scientific background and rationale for the investigation | Introduction |
| 3 | State specific objectives, including any prespecified hypotheses | Introduction |

**Methods**

| Item No. | Recommendation | Reported on Page No. |
| --- | --- | --- |
| 4 | Present key elements of study design early in the paper | Methods (Study Design and Location) |
| 5 | Describe the setting, locations, and relevant dates | Methods (Study Design and Location) |
| 6a | Give eligibility criteria and sources/methods of participant selection | Methods (Population and Sample) |
| 6b | Give matching criteria (if applicable) | Not applicable |
| 7 | Clearly define all outcomes, exposures, predictors, confounders | Methods (Variables) |
| 8 | For each variable, give sources of data and details of measurement | Methods (Data Collection) |
| 9 | Describe any efforts to address potential sources of bias | Methods (Bias statement added in Study Design and Location) |
| 10 | Explain how the study size was arrived at | Methods (Sample size justification) |
| 11 | Explain how quantitative variables were handled (e.g., grouping) | Methods (Variables – age grouping justification) |
| 12a | Describe all statistical methods | Methods (Data Analysis) |
| 12b | Describe methods used to control for confounding | Methods (Logistic regression statement) |
| 12c | Explain how missing data were addressed | Methods (Exclusion of incomplete records) |
| 12d | Describe analytical methods taking account of sampling strategy | Not applicable |
| 12e | Describe any sensitivity analyses | Not applicable |

**Results**

| Item No. | Recommendation | Reported on Page No. |
| --- | --- | --- |
| 13a | Report numbers of individuals at each stage of study | Results (participant flow) |
| 13b | Give reasons for non-participation at each stage | Results (refusal and exclusion explained) |
| 13c | Consider use of a flow diagram | Results (described; figure optional) |
| 14a | Give characteristics of study participants | Results |
| 14b | Indicate number of participants with missing data | Methods (excluded incomplete records) |
| 15 | Report numbers of outcome events or summary measures | Results |
| 16a | Give unadjusted and adjusted estimates with precision (e.g., CI) | Results (tables and regression) |
| 16b | Report category boundaries when continuous variables were categorized | Methods (age grouping clarified) |
| 16c | Consider translating relative risk into absolute risk | Not applicable |
| 17 | Report other analyses (subgroups, interactions) | Results (subgroup and regression analyses) |

**Discussion**

| Item No. | Recommendation | Reported on Page No. |
| --- | --- | --- |
| 18 | Summarize key results with reference to study objectives | Discussion |
| 19 | Discuss limitations, including sources of bias or imprecision | Limitations section |
| 20 | Provide cautious overall interpretation of results | Discussion |
| 21 | Discuss generalisability (external validity) | Discussion (generalisability statement added) |

**Other Information**

| Item No. | Recommendation | Reported on Page No. |
| --- | --- | --- |
| 22 | Give source of funding and role of funders | Funding section |
